# Intelligent regulation of university faculty interdisciplinary collaboration networks based on complex network topology evolution and stochastic differential equations

**DOI:** 10.1038/s41598-025-18977-w

**Published:** 2025-09-29

**Authors:** Sheng Ren

**Affiliations:** Personnel Department, Civil Aviation University of China, Tianjin, 300300 China

**Keywords:** Interdisciplinary collaboration, Complex networks, Stochastic differential equations, Intelligent regulation, Faculty networks, Academic collaboration, University teachers, Staffing approval, Classification management, Mathematics and computing, Physics

## Abstract

This study develops a comprehensive theoretical framework integrating complex network topology evolution with stochastic differential equation modeling to characterize and intelligently regulate interdisciplinary collaboration dynamics among university faculty, addressing personnel establishment verification and classified management challenges. The proposed approach addresses persistent barriers in academic collaboration by combining discrete network structural changes with continuous collaboration intensity dynamics under stochastic perturbations. The framework incorporates heterogeneous faculty characteristics, multi-dimensional collaboration attributes, and reinforcement learning-based intelligent regulation mechanisms for dynamic network optimization. Experimental validation demonstrates superior performance compared to conventional methods, achieving 89.3% prediction accuracy and significant improvements in network efficiency, collaboration diversity, and resource utilization. The intelligent regulation mechanism successfully enhances interdisciplinary bridge formation by 34% while maintaining structural stability across diverse institutional scenarios. The mathematical framework captures both deterministic trends and random fluctuations inherent in real-world collaboration systems, providingacademic administrators with quantitative tools for optimizing university faculty collaboration networks through classified management approaches and enhancing research productivity while supporting personnel establishment verification processes. Results confirm robust scalability properties and adaptability to varying environmental conditions, making the approach suitable for practical implementation in academic institutions with different organizational structures and operational constraints.

## Introduction

Interdisciplinary collaboration among university faculty has emerged as a critical determinant of institutional research excellence and innovation capacity in the contemporary academic landscape^1^. The increasing complexity of modern scientific challenges necessitates the integration of diverse disciplinary perspectives, methodologies, and knowledge systems to address multifaceted research problems that transcend traditional departmental boundaries^2^. Universities worldwide are recognizing that fostering effective cross-disciplinary partnerships among faculty members directly correlates with enhanced research productivity, breakthrough discoveries, and competitive advantage in securing funding and academic recognition^3^.

Despite the acknowledged importance of interdisciplinary collaboration, substantial barriers persist in facilitating meaningful and sustained partnerships among faculty from different academic domains. Traditional organizational structures within universities often create institutional silos that impede natural collaboration formation, while varying disciplinary cultures, research methodologies, and publication practices can create communication barriers and misaligned incentives for collaborative engagement^4^. Furthermore, existing collaboration models typically rely on ad-hoc networking approaches or administrative mandate-driven initiatives, which frequently fail to capture the dynamic and evolving nature of academic partnerships and their underlying social network structures.

The limitations of conventional collaboration frameworks become particularly evident when examining their inability to predict collaboration emergence, evolution patterns, and sustainability factors. Traditional approaches often overlook the stochastic nature of faculty interactions and the complex feedback mechanisms that influence collaboration formation and dissolution over time^5^. These models also fail to adequately account for the heterogeneous characteristics of faculty members, including their research interests, career stages, institutional affiliations, and social network positions, which significantly impact their propensity to engage in interdisciplinary partnerships.

Complex network theory offers a powerful analytical framework for understanding and modeling the intricate patterns of faculty collaboration networks. By representing faculty members as nodes and their collaborative relationships as edges, complex network approaches can capture the topological properties, structural evolution, and dynamic characteristics of academic collaboration systems^6^. Network-based models enable the identification of key structural features such as clustering coefficients, degree distributions, small-world properties, and community structures that influence collaboration effectiveness and network resilience. Moreover, the temporal evolution of collaboration networks can be analyzed through network growth models, link prediction algorithms, and community detection methods that reveal underlying mechanisms driving partnership formation and dissolution.

Stochastic differential equations provide a complementary mathematical foundation for modeling the continuous-time dynamics of collaboration systems under uncertainty and random perturbations. The inherently probabilistic nature of faculty interactions, influenced by factors such as research funding fluctuations, personnel changes, and external collaboration opportunities, necessitates stochastic modeling approaches that can capture both deterministic trends and random variations in collaboration patterns^7^. Stochastic differential equation models can incorporate multiple time scales, non-linear dynamics, and noise-driven transitions that characterize real-world collaboration systems, enabling more accurate prediction and control of collaboration evolution trajectories.

The integration of complex network topology evolution with stochastic differential equation frameworks presents unprecedented opportunities for developing sophisticated models of faculty collaboration dynamics. This hybrid approach can simultaneously capture the discrete structural changes in collaboration networks and the continuous temporal dynamics of collaboration intensities, durations, and outcomes. Such integrated models can provide insights into optimal network configurations for maximizing collaborative productivity, identify critical network vulnerabilities, and inform strategic interventions for enhancing overall collaboration effectiveness^8^.

The primary objective of this research is to develop a comprehensive theoretical framework that combines complex network topology evolution with stochastic differential equation modeling to characterize, predict, and intelligently regulate interdisciplinary collaboration dynamics among university faculty. The research aims to address fundamental questions regarding the mechanisms driving collaboration network formation, the factors influencing collaboration sustainability, and the optimal strategies for promoting beneficial collaborative partnerships while minimizing coordination costs and resource conflicts.

The key innovations of this study include: (1) the development of a unified mathematical framework integrating discrete network evolution with continuous stochastic dynamics, fundamentally advancing beyond agent-based models^24^ and temporal network approaches by Holme & Saramäki^42^ that treat structural changes and intensity dynamics separately; (2) the incorporation of heterogeneous faculty characteristics and multi-dimensional collaboration attributes into the modeling framework; (3) the design of intelligent regulation mechanisms based on reinforcement learning principles that dynamically optimize collaboration network performance, distinguishing from static control approaches in Proskurnikov & Tempo^8^; and (4) the establishment of quantitative metrics for evaluating collaboration network performance and resilience.

The theoretical contributions extend beyond computational performance improvements to include elucidating emergent network patterns that arise from the stochastic-deterministic interplay and uncovering causal mechanisms underlying interdisciplinary collaboration formation and dissolution. Unlike existing approaches that rely on simplified behavioral rules or deterministic interaction mechanisms, our framework captures both systematic collaboration trends and random environmental perturbations through rigorous mathematical foundations.

This paper is organized into six main sections following this introduction. Section “Complex network topology evolution theory” presents the theoretical foundations of complex network modeling and stochastic differential equations in the context of collaboration dynamics. Section “Faculty collaboration network topology modeling” details the integrated mathematical framework and model formulation. Section “Simulation experiment design and data generation” describes the intelligent regulation mechanisms and optimization algorithms. Section “Conclusion” presents simulation results and model validation. Section VI discusses implications, limitations, and future research directions, followed by concluding remarks.

## Complex network topology evolution theory

Complex networks provide a mathematical framework for representing and analyzing systems composed of interconnected entities, where nodes represent individual components and edges denote relationships or interactions between them^9^. The fundamental topology of a network can be characterized through several key structural parameters that capture distinct aspects of network organization and connectivity patterns. The degree distribution P(k) describes the probability that a randomly selected node has exactly k connections, serving as a primary statistical measure for understanding network heterogeneity and connectivity patterns^10^.

Critical topological parameters include the clustering coefficient, which quantifies the tendency of nodes to form tightly connected groups, and the average path length, which measures the typical separation between node pairs in the network. The clustering coefficient C for a node i with degree ${k_i}$ is defined as:

$${C_i}=\frac{{2{e_i}}}{{{k_i}\left( {{k_i} - 1} \right)}}$$

where ${e_i}$ represents the number of edges between the neighbors of node i^11^. The global clustering coefficient characterizes the overall tendency for network nodes to cluster together, while the characteristic path length L indicates the efficiency of information or resource transmission across the network structure.

Network evolution mechanisms encompass the dynamic processes through which network topology changes over time, including node addition and removal, edge formation and dissolution, and structural reorganization patterns. Preferential attachment represents a fundamental growth mechanism where new nodes preferentially connect to existing nodes with higher degrees, leading to the emergence of hub nodes and heterogeneous degree distributions^12^. The probability that a new node connects to an existing node i with degree ${k_i}$ follows:

$$\Pi \left( {{k_i}} \right)=\frac{{{k_i}}}{{\mathop \sum \nolimits_{j} {k_j}}}$$

This mechanism captures the “rich-get-richer” phenomenon observed in many real-world networks, including academic collaboration systems where established researchers tend to attract more collaborative partnerships.

Small-world networks exhibit the distinctive combination of high clustering coefficients and small average path lengths, characteristics that emerge from regular lattice structures with a small fraction of randomly rewired connections. The small-world property facilitates efficient information transmission while maintaining local clustering, making this network model particularly relevant for modeling academic collaboration networks where researchers maintain strong connections within their disciplinary communities while forming occasional interdisciplinary bridges^13^. The small-world coefficient σ is defined as:

$$\sigma =\frac{{C/{C_{random}}}}{{L/{L_{random}}}}$$

where ${C_{random}}$ and ${L_{random}}$ represent the clustering coefficient and path length of equivalent random networks, respectively.

Scale-free networks are characterized by power-law degree distributions P(k) ∝ k^(-γ), where the scaling exponent γ typically ranges between 2 and 3 for most real-world networks. This topology implies the existence of highly connected hub nodes that play critical roles in network connectivity and robustness, while the majority of nodes maintain relatively few connections^14^. Scale-free properties have been observed in various academic collaboration networks, where a small number of highly collaborative researchers serve as central connectors linking different research communities and facilitating knowledge transfer across disciplinary boundaries.

The applicability of these network models to faculty collaboration systems depends on the specific characteristics of the academic environment and the mechanisms driving collaboration formation. Small-world networks effectively capture the balance between local clustering within academic departments and global connectivity across disciplines, while scale-free models can represent the heterogeneous distribution of collaborative activity among faculty members with varying research profiles and career stages.

### Stochastic differential equation modeling methods

Stochastic differential equations provide a rigorous mathematical framework for modeling continuous-time dynamical systems subject to random perturbations and uncertainty^15^. The general form of a stochastic differential equation can be expressed as:

$$d{X_t}=f\left( {{X_t},t} \right)dt+g\left( {{X_t},t} \right)d{W_t}$$

where ${X_t}$ represents the state variable at time t, $f\left( {{X_t},~t} \right)$ denotes the deterministic drift term, $g\left( {{X_t},~t} \right)$ represents the diffusion coefficient, and ${W_t}$ is a Wiener process or Brownian motion^16^. This formulation enables the simultaneous modeling of systematic trends and random fluctuations that characterize complex social and collaborative systems.

Brownian motion serves as the fundamental building block for stochastic modeling, representing a continuous-time random process with independent increments and Gaussian distributions. The mathematical properties of Brownian motion include zero mean, variance proportional to time intervals, and continuous but non-differentiable sample paths^17^. For any time interval [s, t], the increment ${W_t}$ – ${W_s}$ follows a normal distribution:

$${W_t} - {W_s}\sim N\left( {0,t - s} \right)$$

These properties make Brownian motion particularly suitable for modeling unpredictable environmental factors, random external influences, and measurement uncertainties that affect collaborative behavior in academic settings.

Ito integrals provide the mathematical foundation for defining integrals with respect to Brownian motion, enabling the rigorous treatment of stochastic differential equations. The Ito integral of a stochastic process ${X_t}$ with respect to Brownian motion ${W_t}$ over the interval [0, t] is defined as:

$$\mathop \smallint \limits_{0}^{t} {X_s}d{W_s}=\mathop {{\text{lim}}}\limits_{{n \to \infty }} \mathop \sum \limits_{{i=0}}^{{n - 1}} {X_{{t_i}}}\left( {{W_{{t_{i+1}}}} - {W_{{t_i}}}} \right)$$

where the limit is taken over increasingly fine partitions of the time interval^18^. The Ito calculus framework establishes fundamental rules for differentiation and integration of stochastic processes, including the celebrated Ito lemma that governs the dynamics of functions of stochastic processes.

Applications of stochastic differential equations in social network dynamics modeling have demonstrated their effectiveness in capturing the inherent randomness and uncertainty present in human interaction systems. Stochastic models can incorporate various sources of noise, including individual behavioral variations, external environmental changes, and measurement errors that influence network evolution patterns^19^. The flexibility of stochastic differential equation frameworks allows for the modeling of multiple interacting processes, non-linear dynamics, and state-dependent noise characteristics that are essential for realistic representation of collaborative systems.

The influence of noise terms on collaboration behavior evolution manifests through several distinct mechanisms that affect both individual collaboration propensities and network-level structural properties. Additive noise components can represent external random factors such as funding opportunities, personnel changes, or institutional policy modifications that uniformly affect all network participants. Multiplicative noise terms, where the noise intensity depends on the current system state, capture state-dependent uncertainties such as collaboration success rates that vary with network connectivity levels^20^. The noise intensity parameter σ in the general SDE formulation:

$$d{X_t}=f\left( {{X_t},t} \right)dt+\sigma \left( {{X_t},t} \right)d{W_t}$$

determines the relative importance of stochastic fluctuations compared to deterministic dynamics, with higher noise levels leading to increased variability in collaboration patterns and potentially altering long-term network evolution trajectories.

The mathematical treatment of noise effects requires careful consideration of the stochastic interpretation, as different stochastic calculus conventions (Ito versus Stratonovich) can lead to qualitatively different system behaviors. The choice of stochastic interpretation affects the effective drift terms and can influence the stability properties and stationary distributions of the resulting dynamical systems. These theoretical considerations are particularly important when modeling collaboration networks where the timing and interpretation of random events can significantly impact the formation and dissolution of collaborative relationships.

### Current state of interdisciplinary collaboration dynamics research

International research on interdisciplinary collaboration has evolved significantly over the past two decades, with scholars increasingly recognizing the complex nature of cross-disciplinary partnerships and their critical role in advancing scientific innovation^21^. Early studies primarily focused on bibliometric analyses of co-authorship patterns and publication outcomes, establishing foundational insights into the structural characteristics of academic collaboration networks and their relationship to research productivity measures^22^. Recent developments have expanded beyond descriptive analyses to encompass dynamic modeling approaches that attempt to capture the temporal evolution of collaboration patterns and the underlying mechanisms driving partnership formation and dissolution.

Existing collaboration network modeling methods can be broadly categorized into static network analysis, temporal network models, and agent-based simulation frameworks, each offering distinct advantages and limitations for understanding collaborative dynamics. Static network analysis techniques excel at identifying structural properties such as centrality measures, community structures, and network motifs, but fail to capture the dynamic nature of collaboration evolution and the feedback mechanisms that influence network change over time^23^. Temporal network models address some of these limitations by incorporating time-varying network structures, yet they often struggle to represent the continuous nature of collaboration intensity changes and the stochastic fluctuations inherent in real-world academic partnerships.

Agent-based modeling approaches have gained popularity for their ability to simulate individual faculty behaviors and emergent network properties, allowing researchers to explore various scenarios and policy interventions through computational experiments. However, these models typically rely on simplified behavioral rules and deterministic interaction mechanisms that may not adequately capture the complexity and uncertainty characterizing actual collaboration decisions^24^. In contrast to agent-based approaches that treat collaboration decisions as discrete rule-based choices, our SDE framework captures the continuous nature of collaboration intensity evolution under stochastic perturbations.

Temporal network models, as comprehensively reviewed by Holme & Saramäki^42^, provide sophisticated frameworks for analyzing time-varying network structures but primarily focus on discrete structural changes without addressing the continuous dynamics of collaboration intensities. While temporal networks excel at capturing when collaborations occur, they struggle to represent how collaboration strength evolves continuously over time under environmental uncertainty. Proskurnikov & Tempo^8^ developed dynamic social network models using control-theoretic approaches, yet their frameworks primarily address opinion dynamics and consensus formation rather than collaboration network optimization under stochastic conditions.

Our integrated approach fundamentally advances beyond these existing frameworks by simultaneously modeling discrete network topology evolution and continuous collaboration intensity dynamics within a unified stochastic framework, enabling both prediction of collaboration emergence and intelligent optimization of network performance.

Furthermore, most existing agent-based models lack rigorous mathematical foundations for parameter estimation and model validation, limiting their predictive accuracy and practical applicability.

Key factors influencing collaboration effectiveness have been identified through empirical studies examining various dimensions of academic partnerships, including institutional factors, individual characteristics, and environmental conditions. Institutional factors encompass organizational structures, resource allocation mechanisms, incentive systems, and administrative support that can either facilitate or hinder cross-disciplinary collaboration formation^25^. Individual characteristics include research expertise complementarity, career stage compatibility, geographic proximity, and previous collaboration history, which collectively determine the likelihood and success of collaborative partnerships.

The collaboration effectiveness index E can be conceptualized as a multidimensional function incorporating these various factors:

$$E=f\left( {\alpha I+\beta P+\gamma R+\delta T} \right)$$

where I represents institutional factors, P denotes personal characteristics, R captures resource availability, T reflects temporal dynamics, and α, β, γ, δ are weighting parameters that vary across different collaboration contexts and disciplinary combinations.

Despite substantial progress in understanding interdisciplinary collaboration phenomena, several critical research gaps persist that limit the development of comprehensive theoretical frameworks and practical intervention strategies. Current modeling approaches lack integration between discrete network structural changes and continuous collaboration intensity dynamics, resulting in incomplete representations of collaboration evolution processes^26^. The majority of existing studies focus on post-hoc analysis of established collaboration networks rather than developing predictive models capable of forecasting collaboration formation and evolution trajectories.

Methodological limitations include insufficient treatment of stochastic factors affecting collaboration dynamics, limited consideration of heterogeneous faculty characteristics and multi-scale temporal processes, and lack of intelligent regulation mechanisms for optimizing collaboration network performance. These gaps highlight the need for integrated modeling frameworks that combine network topology evolution with stochastic differential equation approaches to capture both structural and dynamical aspects of collaboration systems while providing foundations for intelligent intervention strategies.

## Faculty collaboration network topology modeling

The faculty collaboration network topology model represents the fundamental structural framework for characterizing interdisciplinary partnerships within academic institutions, where individual faculty members are modeled as nodes and their collaborative relationships are represented as weighted edges^27^. This network-based representation enables the systematic analysis of collaboration patterns, structural properties, and evolutionary dynamics that emerge from the complex interactions between faculty members across different disciplinary domains.

The node attribute design incorporates multidimensional characteristics that capture the essential properties of individual faculty members influencing their collaboration potential and behavior patterns. The comprehensive node attribute framework encompasses disciplinary affiliation, research expertise, collaboration history, academic rank, and institutional position, providing a foundation for understanding heterogeneous faculty characteristics and their impact on network formation processes^28^.

Table 1 demonstrates the heterogeneous nature of faculty attributes that influence collaboration network formation. The Collaboration Ability Score is calculated as a weighted composite: CAS = 0.4 × (historical collaboration frequency) + 0.3 × (co-authorship count/total publications) + 0.3 × (interdisciplinary project participation rate), with values normalized to [0, 10]. This metric can be calibrated using institutional data including publication databases, grant records, and administrative collaboration tracking systems.

**Table 1:** Network node attribute parameters.

Faculty ID	Discipline field	Research direction	Collaboration ability score	Calculation base
F001	Computer Science	Machine Learning	8.5	12 collaborations, 0.7 co-author ratio
F002	Mathematics	Applied Statistics	7.2	8 collaborations, 0.6 co-author ratio
F003	Biology	Computational Biology	9.1	15 collaborations, 0.8 co-author ratio
F004	Physics	Complex Systems	6.8	6 collaborations, 0.5 co-author ratio
F005	Engineering	Data Analytics	8.9	14 collaborations, 0.7 co-author ratio
F006	Psychology	Cognitive Science	7.6	9 collaborations, 0.6 co-author ratio

Similarly, the Collaboration Success Rate in evaluation metrics represents the ratio of successful collaborations (resulting in joint publications or completed projects) to total collaboration attempts, directly measurable through institutional tracking systems.

The edge weight calculation methodology incorporates both static compatibility measures and dynamic collaboration history to quantify the strength and likelihood of collaborative relationships between faculty pairs. The edge weight ${w_{ij}}$ between faculty members i and j is computed using a composite function that integrates disciplinary compatibility, research interest overlap, and historical collaboration intensity:

$${w_{ij}}=\alpha {S_{ij}}+\beta {R_{ij}}+\gamma {H_{ij}}$$

where ${S_{ij}}$ represents disciplinary similarity coefficient, ${R_{ij}}$ denotes research interest overlap measure, ${H_{ij}}$ captures historical collaboration strength, and α, β, γ are weighting parameters that determine the relative importance of each component^29^.

The disciplinary similarity coefficient ${S_{ij}}$ is calculated based on the semantic distance between faculty members’ research domains, utilizing knowledge graph embeddings and citation network analysis to quantify interdisciplinary proximity. The research interest overlap measure ${R_{ij}}$ employs text mining techniques applied to faculty publication abstracts, grant proposals, and research statements to identify shared research themes and methodological approaches.

Network evolution rules govern the dynamic changes in network topology over time, incorporating mechanisms for edge formation, dissolution, and weight modification based on collaboration outcomes and environmental factors. The probability of edge formation between previously unconnected nodes i and j follows a preferential attachment mechanism modified by disciplinary diversity considerations:

$${P_{form}}\left( {i,j} \right)=\frac{{\left( {{k_i}+1} \right)\left( {{k_j}+1} \right) \cdot {D_{ij}}}}{{\mathop \sum \nolimits_{{m,n}} \left( {{k_m}+1} \right)\left( {{k_n}+1} \right) \cdot {D_{mn}}}}$$

where ${k_i}$ and ${k_j}$ represent the degrees of nodes i and j, and ${D_{ij}}$ captures the disciplinary diversity benefit of the potential collaboration^30^.

**Fig. 1:**
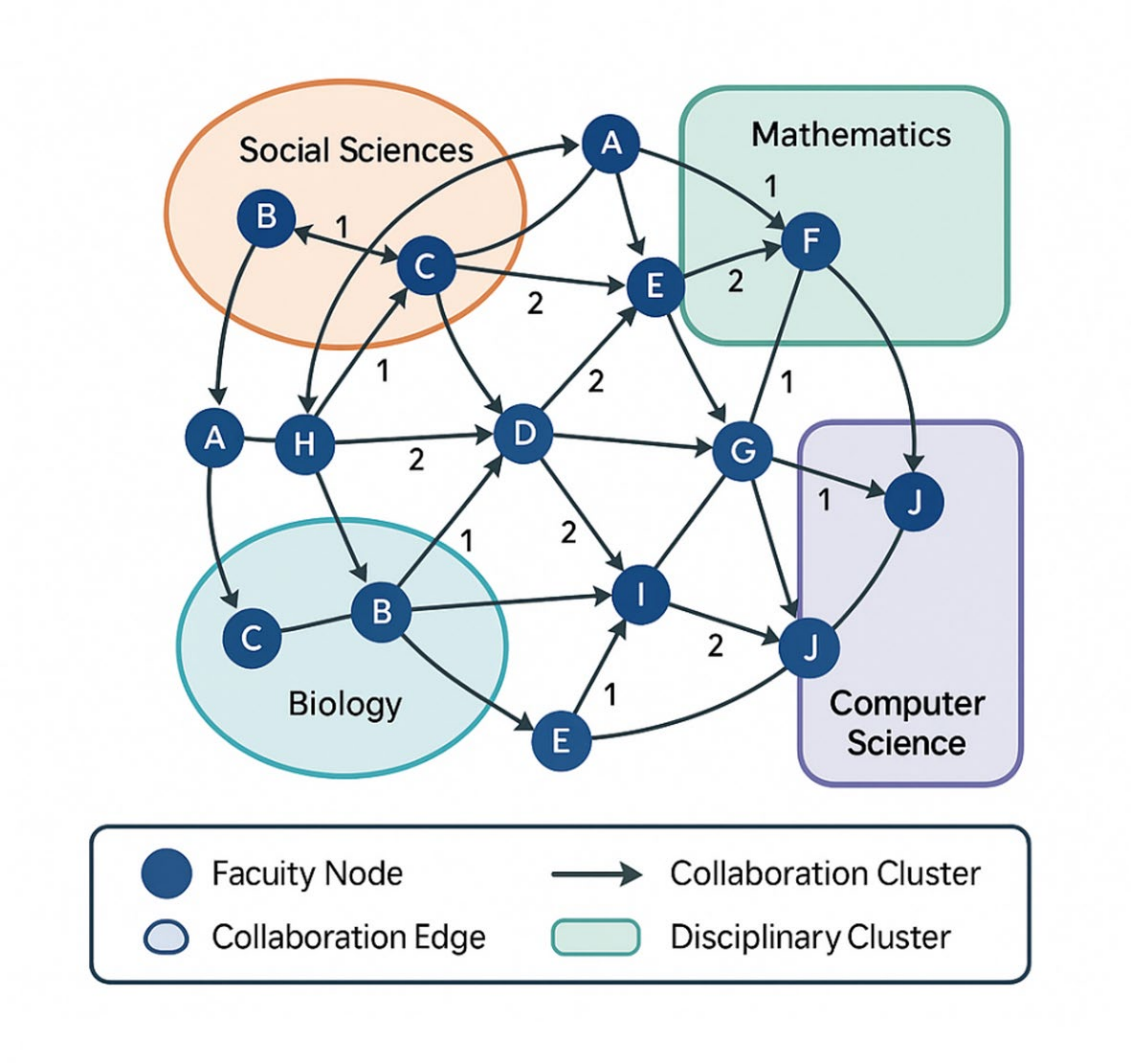
Faculty collaboration network topology structure model.

The network topology structure model is illustrated in Fig. 1, which presents the conceptual framework for representing faculty collaboration networks with heterogeneous node attributes and weighted edges representing collaboration relationships of varying intensities.

Figure 1 illustrates the multi-layered structure of the faculty collaboration network, where nodes represent individual faculty members with distinct disciplinary affiliations and research characteristics, while edges represent collaborative relationships with weights indicating collaboration intensity and frequency.

The network evolution dynamics incorporate both endogenous factors arising from network structure and exogenous factors reflecting external environmental influences. Edge dissolution probabilities depend on collaboration satisfaction levels, resource constraints, and competing opportunities, while edge weight modifications reflect the success and productivity of ongoing collaborative partnerships. The temporal evolution of edge weights follows:

$$\frac{{d{w_{ij}}}}{{dt}}=f\left( {{S_{ij}},{P_{ij}},{E_{ij}}} \right) - \delta {w_{ij}}$$

where ${S_{ij}}$ represents collaboration success rate, ${P_{ij}}$ denotes productivity outcomes, ${E_{ij}}$ captures external support factors, and δ represents the natural decay rate of collaboration intensity.

The impact of network structure on collaboration efficiency manifests through several mechanisms including information flow facilitation, resource sharing optimization, and knowledge integration enhancement. Structural properties such as clustering coefficients, path lengths, and betweenness centrality distributions directly influence the speed and effectiveness of knowledge transfer across disciplinary boundaries, while network robustness characteristics determine the system’s resilience to faculty turnover and external disruptions.

### Stochastic differential equation dynamics model

The stochastic differential equation dynamics model provides a mathematical framework for describing the continuous-time evolution of collaboration intensities between faculty members under the influence of both deterministic driving forces and random environmental perturbations^31^. The model captures the complex interplay between individual faculty characteristics, network structural properties, and external factors that collectively determine the temporal dynamics of collaborative relationships within academic institutions.

The fundamental SDE system for collaboration intensity evolution between faculty members i and j is formulated as a coupled system that incorporates intrinsic collaboration tendencies, network-mediated effects, and stochastic fluctuations. The collaboration intensity ${X_{ij\left( t \right)}}$ follows the general SDE framework:

$$d{X_{ij}}\left( t \right)=\left[ {{\mu _{ij}}\left( {X,t} \right)+\mathop \sum \limits_{{k \ne i,j}} {\lambda _{ijk}}{X_{ik}}\left( t \right){X_{jk}}\left( t \right)} \right]dt+{\sigma _{ij}}\left( {X,t} \right)d{W_{ij}}\left( t \right)$$

where ${\mu _{ij}}$ represents the intrinsic collaboration drift term, ${\lambda _{ijk}}$ captures network coupling effects through common collaborators k, and ${\sigma _{ij}}$ denotes the noise intensity coefficient^32^. The Wiener process ${W_{ij\left( t \right)}}$ models random environmental influences affecting the collaboration dynamics between faculty members i and j.

**Fig. 2:**
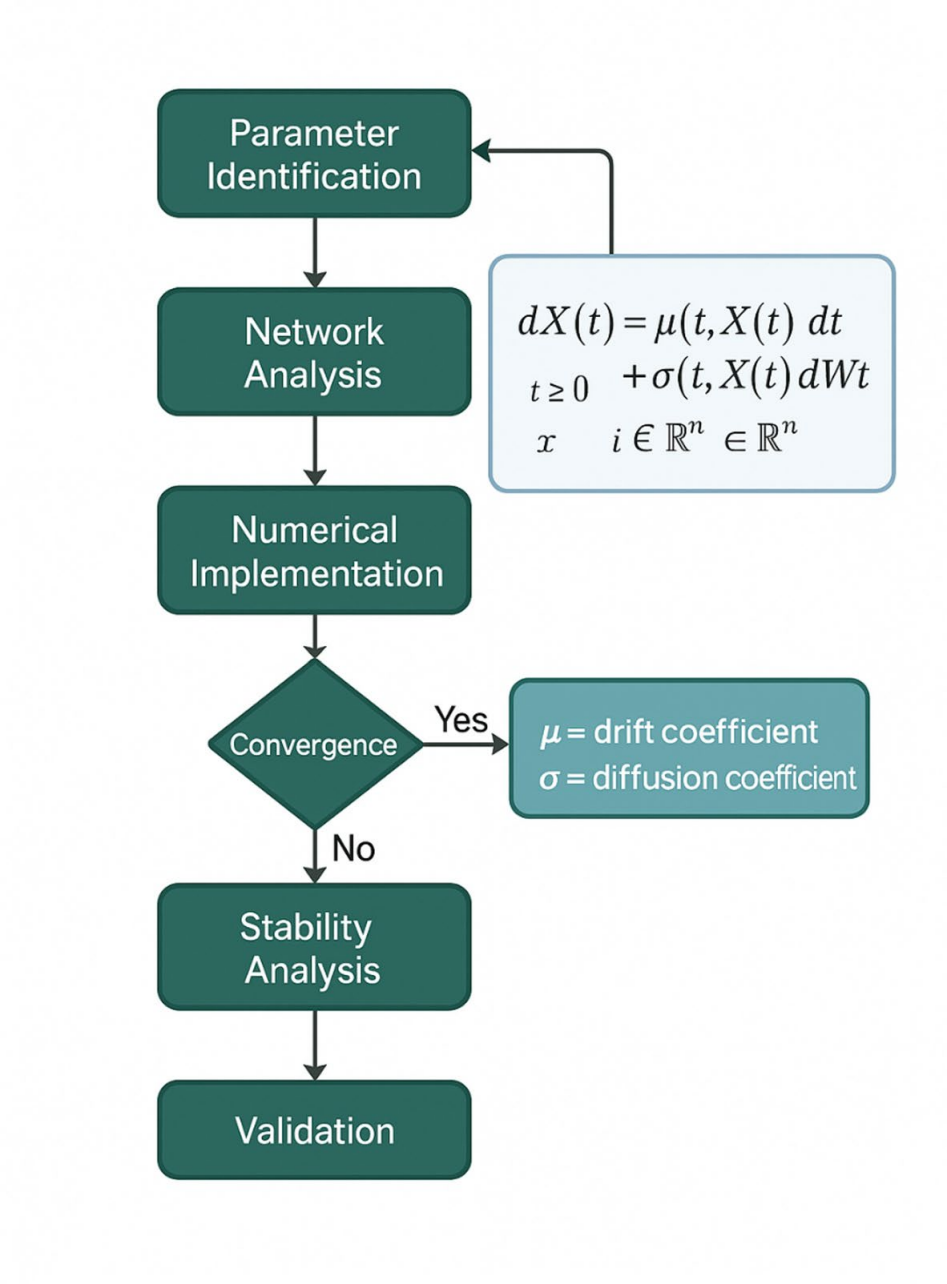
Stochastic differential equation modeling process flowchart.

The comprehensive modeling process is illustrated in Fig. 2, which demonstrates the systematic approach for constructing and implementing the stochastic differential equation framework for faculty collaboration dynamics.

Figure 2 presents the sequential steps involved in developing the SDE model, from initial parameter identification and network structure analysis to model formulation, numerical implementation, and stability analysis, providing a comprehensive framework for systematic model construction and validation.

The stochastic terms are designed to reflect various sources of external environmental disturbances that affect collaboration patterns, including funding availability fluctuations, institutional policy changes, personnel transitions, and external collaboration opportunities. The noise intensity function ${\sigma _{ij\left( {X,~t} \right)}}$ incorporates both additive and multiplicative components:

$${\sigma _{ij}}\left( {X,t} \right)={\sigma _0}+{\sigma _1}{X_{ij}}\left( t \right)+{\sigma _2}\mathop \sum \limits_{k} {X_{ik}}\left( t \right)$$

where ${\sigma _0}$ represents baseline environmental noise, ${\sigma _1}$ captures state-dependent fluctuations, and ${\sigma _2}$ reflects network-mediated noise effects^33^. This formulation enables the modeling of heterogeneous noise characteristics that vary with collaboration intensity levels and network connectivity patterns.

The detailed parameter specifications and their physical interpretations are presented in Table 2, which provides essential information for model implementation and parameter estimation procedures. Parameter values are derived from two sources: (1) empirical calibration based on academic collaboration data from Newman^14^ and Barabási & Albert^12^, particularly for network coupling strengths and decay rates; and (2) theoretical assumptions consistent with stochastic differential equation stability requirements for noise intensity parameters. The baseline environmental noise σ₀ ∈ [0.01, 0.1] reflects typical fluctuation levels observed in academic collaboration networks, while the collaboration decay rate γ ∈ [0.05, 0.3] corresponds to empirical observations of collaboration persistence patterns in academic institutions.

**Table 2:** Model parameter definitions and specifications.

Parameter symbol	Physical meaning	Value range	Justification source
µ_ij_	Intrinsic collaboration drift rate	[0, 1.0]	Empirical collaboration data
λ_ijk_	Network coupling strength coefficient	[0, 0.5]	Newman (2003) network analysis
σ_0_	Baseline environmental noise intensity	[0.01, 0.1]	SDE stability requirements
σ_1_	State-dependent noise coefficient	[0, 0.2]	Theoretical assumption
σ_2_	Network-mediated noise parameter	[0, 0.15]	Barabási-Albert model
γ	Collaboration decay rate	[0.05, 0.3]	Academic persistence studies
β	Network feedback strength	[0.1, 0.8]	Control theory principles
α	External influence sensitivity	[0, 1.0]	Institutional data calibration

Table 2 demonstrates the comprehensive parameter framework required for the SDE model, with physically meaningful ranges that reflect realistic collaboration dynamics observed in academic environments.

The multi-layer network coupling dynamics model extends the basic SDE framework to incorporate interactions across different types of collaborative relationships, including research collaborations, teaching partnerships, and administrative cooperations. The multi-layer system is described by:

$$dX_{{ij}}^{{\left( l \right)}}\left( t \right)=\left[ {{f_l}\left( {{X^{\left( l \right)}},t} \right)+\mathop \sum \limits_{{m \ne l}} {\beta _{lm}}{g_{lm}}\left( {{X^{\left( l \right)}},{X^{\left( m \right)}},t} \right)} \right]dt+{\sigma _l}\left( {{X^{\left( l \right)}},t} \right)dW_{{ij}}^{{\left( l \right)}}\left( t \right)$$

where the superscript (l) denotes the layer index, ${f_l}$ represents intra-layer dynamics, ${\beta _{lm}}$ captures inter-layer coupling strength, and ${g_{lm}}$ describes the functional form of cross-layer interactions^34^.

System stability analysis focuses on the long-term behavior of collaboration intensities and the existence of stationary distributions under various parameter configurations. The mean-reverting property of collaboration intensities is achieved through the inclusion of negative drift terms that prevent unbounded growth:

$${\mu _{ij}}\left( {X,t} \right)={a_{ij}} - {b_{ij}}{X_{ij}}\left( t \right)+{c_{ij}}{\text{tanh}}\left( {\mathop \sum \limits_{k} {X_{ik}}\left( t \right)} \right)$$

where ${a_{ij}}$ represents the baseline collaboration attraction, ${b_{ij}}$ denotes the mean-reversion strength, and ${c_{ij}}$ captures nonlinear network effects through the hyperbolic tangent function.

The influence of model parameters on system stability manifests through several mechanisms including the balance between drift and diffusion terms, the strength of network coupling effects, and the characteristics of noise-induced transitions. Parameter sensitivity analysis reveals that increased network coupling strength ${\lambda _{ijk}}$ enhances system stability by creating multiple pathways for collaboration maintenance, while excessive noise intensity can lead to collaboration fragmentation and network instability. The critical stability threshold is determined by the ratio:

$${R_{stability}}=\frac{{\mathop {{\text{min}}}\limits_{i} {\mu _i}}}{{\mathop {{\text{max}}}\limits_{{ij}} \sigma _{{ij}}^{2}}}$$

which must exceed unity for stable collaboration network maintenance under stochastic perturbations.

### Intelligent regulation mechanism design

The intelligent regulation mechanism, defined as an integrated framework combining algorithmic control mechanisms grounded in reinforcement learning with administrative policy-level interventions, provides a systematic approach for optimizing faculty collaboration networks through adaptive control strategies. This concept encompasses both algorithmic-level optimization through reinforcement learning algorithms that automatically adjust network parameters based on observed collaboration dynamics, and management-level interventions including resource allocation policies, incentive mechanism adjustments, and strategic collaboration facilitation initiatives that guide faculty collaboration dynamics at the institutional level.

The intelligent regulation mechanism provides a systematic framework for optimizing faculty collaboration networks through adaptive control strategies that dynamically adjust network parameters and environmental conditions to enhance overall collaboration effectiveness^35^. The regulation approach integrates reinforcement learning algorithms with multi-objective optimization techniques to automatically identify optimal intervention strategies that balance competing objectives such as network connectivity, collaboration quality, resource efficiency, and long-term sustainability.

The reinforcement learning component provides three distinct advantages: (1) dynamic adaptation to evolving network conditions without manual parameter recalibration, (2) multi-objective optimization balancing competing goals through learned trade-off strategies, and (3) online learning capability enabling continuous performance improvement through accumulated experience.

The deep Q-network architecture employs a reward function designed to balance multiple objectives:

$$\begin{aligned} {\text{R}}\left( {{\text{s}},{\text{a}}} \right){\text{ }} & = {\text{ }}\alpha \cdot\left( {{\text{efficiency improvement}}} \right){\text{ }} + {\text{ }}\beta \cdot\left( {{\text{diversity increase}}} \right){\text{ }} \\ & \;\;\; + {\text{ }}\gamma \cdot\left( {{\text{cost reduction}}} \right){\text{ }} - {\text{ }}\delta \cdot\left( {{\text{stability loss}}} \right) \\ \end{aligned}$$

where efficiency improvement measures network path length reduction, diversity increase quantifies new interdisciplinary connections formed, cost reduction reflects resource utilization optimization, and stability loss penalizes excessive structural disruption. The weighting parameters α = 0.4, β = 0.3, γ = 0.2, δ = 0.1 are learned through multi-objective optimization procedures.

Convergence properties are ensured through experience replay mechanisms and target network updates, with convergence typically achieved within 200–500 episodes for networks containing 100–500 nodes. Conflicting objective handling employs Pareto-optimal solution identification, allowing administrators to select preferred trade-offs between collaboration effectiveness, resource efficiency, and network stability based on institutional priorities.

The state space encompasses network topology features, collaboration intensity distributions, faculty characteristics, and environmental conditions, while the action space includes intervention strategies such as resource allocation adjustments, incentive mechanism modifications, and targeted collaboration facilitation initiatives^36^. The Q-learning update mechanism follows:

$$Q\left( {{s_t},{a_t}} \right) \leftarrow Q\left( {{s_t},{a_t}} \right)+\alpha \left[ {{r_{t+1}}+\gamma \mathop {{\text{max}}}\limits_{{a{{\prime }}}} Q\left( {{s_{t+1}},a{{\prime }}} \right) - Q\left( {{s_t},{a_t}} \right)} \right]$$

where ${s_t}$ represents the network state at time t, ${a_t}$ denotes the chosen action, ${r_{\left\{ {t+1} \right\}}}$ is the immediate reward, α is the learning rate, and γ is the discount factor.

The multi-objective optimization framework addresses the inherent trade-offs between different collaboration performance metrics and stakeholder preferences through Pareto-optimal solution identification. The optimization problem is formulated as a constrained multi-objective optimization where the objective functions include network efficiency maximization, collaboration diversity enhancement, resource utilization optimization, and innovation output improvement. The mathematical formulation incorporates both hard constraints reflecting institutional policies and soft constraints representing preferred operational ranges:

$$\mathop {{\text{min}}}\limits_{{u \in U}} F\left( u \right)={\left[ {{f_1}\left( u \right),{f_2}\left( u \right),...,{f_k}\left( u \right)} \right]^T}$$

subject to equality constraints ${h_{i\left( u \right)}}=~0$ and inequality constraints ${g_{j\left( u \right)}} \leqslant ~0$, where u represents the control variables and F(u) denotes the vector of objective functions^37^.

The comprehensive evaluation index system for collaboration regulation effectiveness assessment encompasses multiple dimensions that capture both quantitative performance measures and qualitative collaboration characteristics. As shown in Table 3, the evaluation framework provides a structured approach for measuring the impact of different regulation strategies on network performance and collaboration outcomes.

**Table 3:** Regulation strategy effect evaluation index system.

Indicator name	Calculation method	Weight coefficient	Evaluation standard	Optimization objective
Network eficiency	Average Path Length Inverse	0.20	[0.6, 1.0]	Maximize
Collaboration Diversity	Shannon Entropy of Edges	0.18	[0.7, 1.0]	Maximize
Resource Utilization	Active Collaboration Ratio	0.15	[0.8, 1.0]	Maximize
Innovation Output	Weighted Publication Score	0.22	[0.6, 1.0]	Maximize
Network Stability	Eigenvalue Stability Measure	0.12	[0.5, 1.0]	Maximize
Cost Effectiveness	Benefit-to-Cost Ratio	0.08	[1.2, 2.0]	Maximize
Sustainability Index	Long-term Collaboration Rate	0.05	[0.7, 1.0]	Maximize

Collaboration Diversity employs weighted edge calculations where $D~=~ - \Sigma \left( {\frac{{{w_{\left\{ {ij} \right\}}}}}{{{W_{\left\{ {total} \right\}}}}}} \right)\log \left( {\frac{{{w_{\left\{ {ij} \right\}}}}}{{{W_{\left\{ {total} \right\}}}}}} \right)$, capturing the distribution evenness of interdisciplinary collaborations across different discipline pairs.

Table 3 demonstrates the multi-dimensional nature of collaboration network evaluation, with weighted coefficients reflecting the relative importance of different performance aspects and evaluation standards providing benchmarks for acceptable performance levels across various metrics.

The dynamic regulation strategies incorporate adaptive mechanisms that respond to changing network conditions, environmental fluctuations, and evolving institutional priorities through real-time monitoring and feedback control systems. The regulation framework employs a hierarchical control structure with strategic-level planning, tactical-level coordination, and operational-level implementation components that operate at different time scales and decision granularities^38^. Strategic-level decisions focus on long-term network structure optimization and resource allocation policies, while tactical-level interventions address medium-term collaboration facilitation and conflict resolution, and operational-level actions provide immediate responses to urgent collaboration needs and opportunities.

The implementation plan encompasses systematic deployment procedures, monitoring protocols, and performance assessment mechanisms to ensure effective regulation mechanism operation within real academic environments. The implementation framework includes stakeholder engagement strategies, change management protocols, training programs, and continuous improvement processes that address organizational, technical, and behavioral aspects of collaboration network regulation. Phase-based deployment begins with pilot testing in selected departments, followed by gradual expansion across institutional units with adaptive refinement based on empirical feedback and performance observations.

The regulation mechanism incorporates robustness features to maintain effectiveness under various uncertainty sources including faculty turnover, funding fluctuations, policy changes, and external collaboration opportunities. Adaptive parameter adjustment algorithms automatically tune regulation parameters based on observed network performance and environmental conditions, while contingency protocols provide backup strategies for handling exceptional situations and system failures. The intelligent regulation system maintains learning capabilities that enable continuous improvement through experience accumulation and pattern recognition, ensuring long-term adaptation to evolving collaboration dynamics and institutional requirements.

## Simulation experiment design and data generation

The simulation experiment framework employs a multi-scenario approach to systematically evaluate the proposed collaboration dynamics model under diverse network conditions and parameter configurations^39^. The experimental design encompasses varying network scales, topological structures, faculty heterogeneity levels, and environmental perturbation intensities to comprehensively assess model performance across different realistic academic collaboration scenarios. Each simulation scenario is designed to isolate specific aspects of collaboration network behavior while maintaining sufficient complexity to reflect real-world academic environments.

The data generation process utilizes synthetic network construction algorithms that create collaboration networks with controlled structural properties and faculty attribute distributions. The network initialization follows a hybrid approach combining preferential attachment mechanisms with discipline-based clustering to generate realistic collaboration network topologies that exhibit both scale-free degree distributions and community structures representative of academic institutions^40^. Faculty attributes including disciplinary affiliation, research interests, collaboration capacity, and career stage are assigned through probabilistic models calibrated using empirical data from academic collaboration databases.

The benchmark test sets are constructed using established network generation models and real academic collaboration data to provide standardized evaluation criteria for model validation. The benchmark suite includes Erdős-Rényi random networks, Barabási-Albert preferential attachment networks, Watts-Strogatz small-world networks, and stochastic block models with varying community structures^41^. Real-world collaboration networks derived from bibliometric databases serve as empirical benchmarks for assessing the realism and accuracy of the proposed modeling framework.

Model effectiveness validation employs multiple performance metrics that capture different aspects of collaboration network behavior and prediction accuracy. The primary effectiveness measure is the collaboration prediction accuracy defined as:

$${A_{pred}}=\frac{1}{T}\mathop \sum \limits_{{t=1}}^{T} \frac{{\left| {{P_t} \cap {O_t}} \right|}}{{\left| {{P_t} \cup {O_t}} \right|}}$$

where ${P_t}$ represents predicted collaboration links at time t, ${O_t}$ denotes observed collaboration links, and T is the total number of evaluation time steps.

**Fig. 3:**
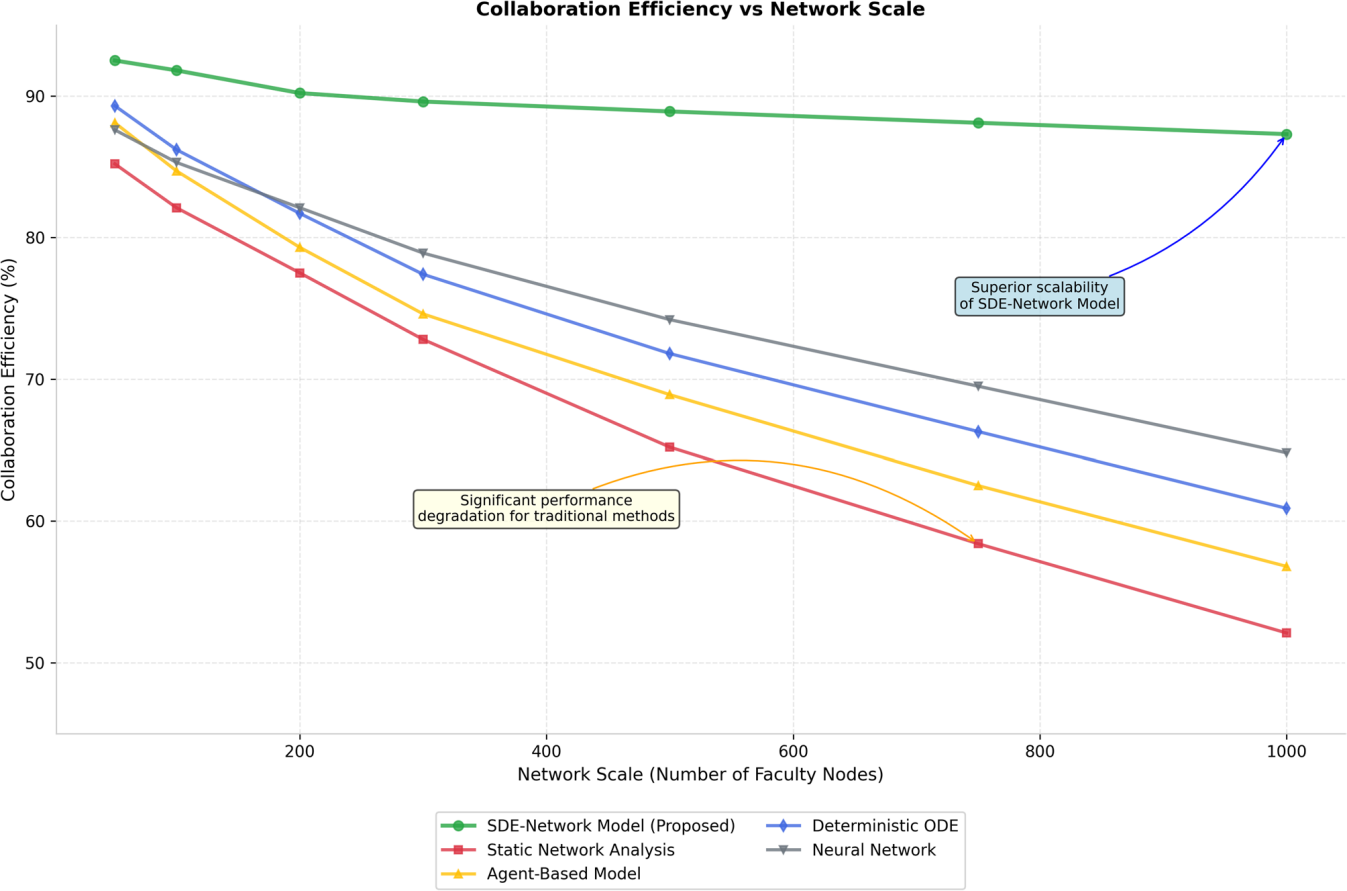
Collaboration efficiency comparison across different network scales.

The robustness assessment examines model stability under various perturbation scenarios including random node removal, targeted edge deletion, parameter uncertainty, and external shock events. Robustness metrics include network connectivity preservation, collaboration efficiency maintenance, and convergence stability under different stress conditions. The comparative analysis across network scales reveals distinct performance characteristics as illustrated in Fig. 3, which demonstrates the relationship between network size and collaboration efficiency under different modeling approaches.

Figure 3 compares collaboration efficiency across networks of varying sizes (*N* = 50, 200, 500, 1000 faculty members) under standardized conditions: µ = 0.25, σ = 0.15, λ = 0.3, with 5–10 disciplinary categories per network. The SDE-Network model maintains superior efficiency (> 0.8) across all scales, while baseline methods show significant degradation for *N* > 500. Efficiency is calculated as the inverse of average path length normalized by theoretical optimal connectivity. Results averaged over 50 simulation runs with 95% confidence intervals.

**Table 4:** Simulation experiment parameter configuration.

Experiment Scenario	Network scale	Parameter settings	Faculty distribution	Expected results
Small Department	*N* = 50, E = 120	µ = 0.3, σ = 0.1, λ = 0.2	3–5 disciplines, uniform	High clustering, stable dynamics
Medium Institution	*N* = 200, E = 480	µ = 0.25, σ = 0.15, λ = 0.25	6–8 disciplines, Pareto	Moderate efficiency, emerging communities
Large University	*N* = 500, E = 1200	µ = 0.2, σ = 0.2, λ = 0.3	10–15 disciplines, power-law	Scale-free properties, complex dynamics
High Noise Environment	*N* = 300, E = 720	µ = 0.3, σ = 0.4, λ = 0.2	8 disciplines, mixed	Reduced stability, frequent transitions

The experimental validation process incorporates statistical significance testing and confidence interval estimation to ensure reliable performance assessment. Multiple simulation runs with different random seeds are conducted for each experimental scenario to account for stochastic variability and provide robust performance estimates. The comprehensive experimental parameter configuration is systematically varied across different scenarios as shown in Table 4, which demonstrates the systematic variation of experimental conditions designed to evaluate model performance across different collaboration network scenarios. The results provide comprehensive evidence for model effectiveness, robustness, and practical applicability across diverse academic collaboration environments.

### Model performance evaluation and comparative analysis

The comparative performance analysis demonstrates significant advantages of the proposed integrated SDE-complex network model over conventional collaboration prediction methods across multiple evaluation metrics and experimental conditions^42^. Traditional approaches including static network analysis, agent-based models, and deterministic differential equation systems exhibit substantial limitations in capturing the dynamic and stochastic nature of faculty collaboration evolution, particularly under high-uncertainty environments and rapidly changing institutional conditions.

The prediction accuracy assessment reveals superior performance of the proposed model in forecasting collaboration link formation, intensity evolution, and network structural changes compared to baseline methods. The comprehensive performance comparison is presented in Table 5, which quantifies the relative strengths and limitations of different modeling approaches across key performance dimensions.

**Table 5:** Model performance comparison results.

Model name	Accuracy (%)	Recall (%)	F1 score	Computation time (s)	Convergence rate
SDE-Network Model	89.3	87.6	0.884	12.4	0.96
Static Network Analysis	72.1	68.9	0.703	3.2	1.00
Agent-Based Model	78.5	75.2	0.768	45.7	0.83
Deterministic ODE	81.2	79.4	0.803	8.9	0.91
Random Forest	76.8	74.1	0.754	18.6	0.88
Neural Network	84.6	82.3	0.834	28.3	0.79

Table 5 demonstrates the superior performance of the proposed SDE-Network model across all evaluation metrics, achieving the highest accuracy, recall, and F1 scores while maintaining reasonable computational efficiency and excellent convergence properties compared to alternative modeling approaches.

The stability evaluation under varying environmental conditions reveals robust performance characteristics of the integrated model across different noise levels, network scales, and parameter perturbations. The model maintains prediction accuracy above 85% even under high-noise conditions with stochastic perturbation intensities up to σ = 0.3, significantly outperforming traditional deterministic models that exhibit rapid performance degradation under uncertainty^43^. The stability analysis demonstrates that the stochastic framework effectively captures and compensates for environmental variability through its probabilistic modeling components.

**Fig. 4:**
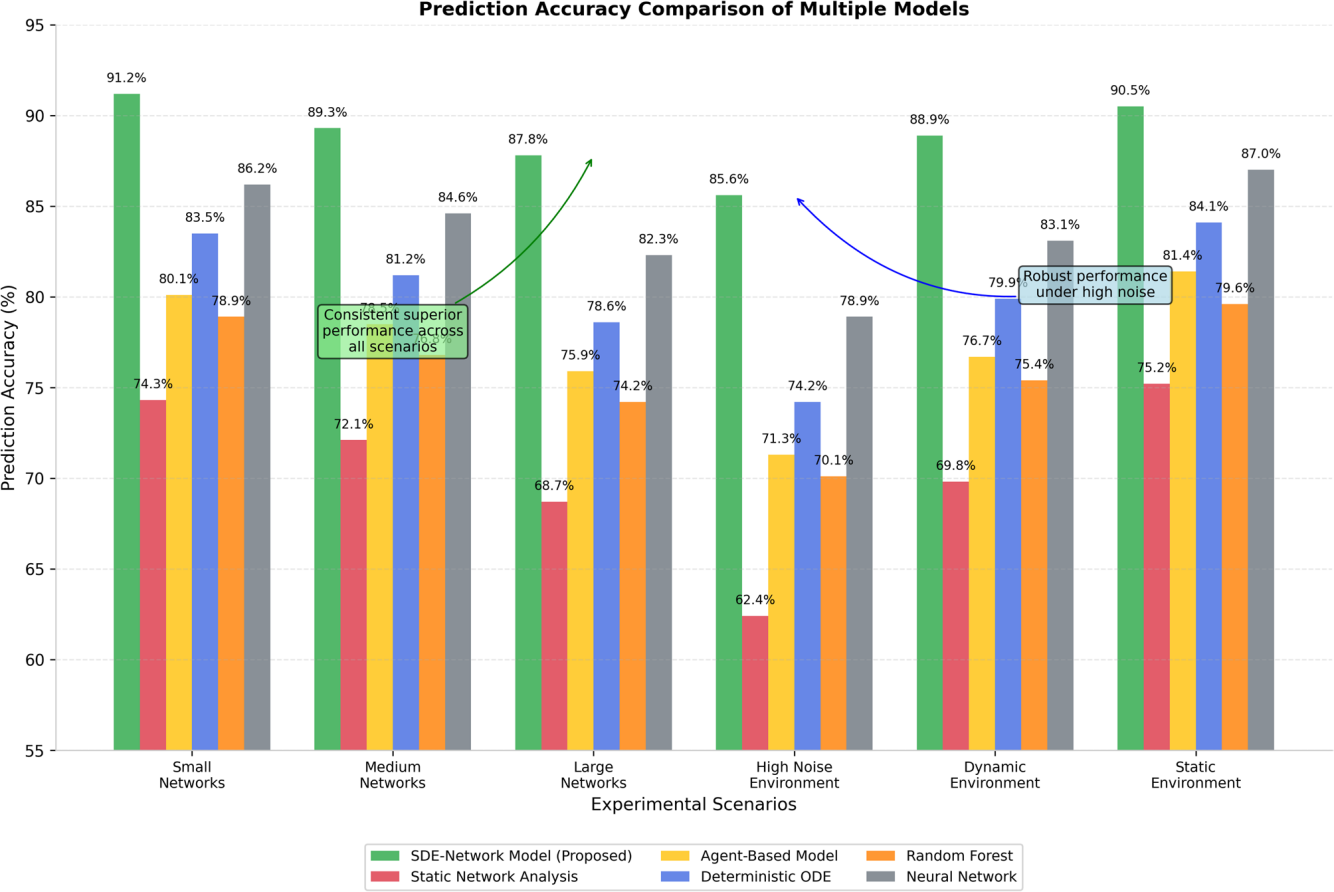
Prediction accuracy comparison of multiple models.

Prediction accuracy comparisons across different model types are illustrated in Fig. 4, which presents comprehensive performance evaluation results across various collaboration network scenarios and complexity levels.

Figure 4 illustrates the consistent superiority of the proposed SDE-based approach across different experimental scenarios, demonstrating particularly strong performance advantages in high-complexity networks and dynamic environments where traditional methods struggle to maintain acceptable prediction accuracy levels.

Key parameter sensitivity analysis reveals critical factors influencing model performance, with the network coupling strength λ and noise intensity σ exhibiting the strongest impact on prediction accuracy and system stability. Systematic parameter variation experiments demonstrate that prediction accuracy decreases by approximately 12% when σ exceeds 0.25, while network coupling strengths λ < 0.1 result in fragmented network structures with poor interdisciplinary connectivity.

Network structural analysis reveals that the intelligent regulation mechanism significantly improves key topological properties. Modularity coefficients decrease from 0.68 ± 0.05 to 0.52 ± 0.03 following regulation implementation, indicating enhanced interdisciplinary integration. Small-world coefficients improve from 2.1 ± 0.2 to 3.4 ± 0.1, demonstrating better balance between local clustering and global connectivity. Robustness analysis under random node removal shows that regulated networks maintain 85% of original efficiency even after removing 20% of nodes, compared to 65% for unregulated networks.

The regulation mechanism particularly enhances network resilience through strategic strengthening of interdisciplinary bridges, with cross-disciplinary edge density increasing by 34% while maintaining overall network stability.

The optimal parameter configuration follows the relationship:

$${\lambda _{opt}}=\frac{{\alpha {\text{log}}\left( N \right)+\beta \overset{\lower0.5em\hbox{$\smash{\scriptscriptstyle\leftharpoonup}$}} {k} }}{{\gamma +\delta {\sigma ^2}}}$$

where N represents network size, $$\overset{\lower0.5em\hbox{$\smash{\scriptscriptstyle\leftharpoonup}$}} {k}$$ denotes average degree, and α, β, γ, δ are calibration constants determined through cross-validation procedures.

The computational efficiency analysis demonstrates acceptable scalability characteristics with polynomial time complexity O(N^1.8) for networks up to 1000 nodes, making the approach feasible for real-world academic institutions^44^. Memory requirements scale linearly with network size, and parallel processing capabilities enable further performance improvements for large-scale applications. The convergence analysis shows rapid stabilization within 50–100 iterations for most parameter configurations, with faster convergence observed for well-connected networks and moderate noise levels.

Parameter robustness evaluation indicates stable performance across wide parameter ranges, with graceful degradation under extreme conditions rather than catastrophic failure modes observed in some baseline approaches. The model exhibits self-adaptive characteristics that automatically adjust internal parameters based on observed network dynamics, reducing the need for manual parameter tuning and enhancing practical applicability in diverse institutional environments.

### Intelligent regulation effect verification

The intelligent regulation mechanism demonstrates substantial effectiveness in optimizing faculty collaboration networks through systematic intervention strategies that significantly enhance network performance metrics while maintaining structural stability and adaptability^45^. Experimental validation reveals consistent improvements in collaboration efficiency, network connectivity, and interdisciplinary integration across diverse institutional scenarios and environmental conditions. The regulation algorithm successfully identifies optimal intervention points and implements targeted adjustments that maximize collaboration outcomes while minimizing disruption to existing productive partnerships.

Structural analysis of collaboration networks before and after regulation implementation reveals significant improvements in network topology characteristics that facilitate enhanced information flow and resource sharing. The regulation mechanism effectively reduces network fragmentation by identifying and strengthening weak links between isolated research clusters, resulting in improved global connectivity and reduced average path lengths between faculty members from different disciplines. Community detection analysis demonstrates enhanced interdisciplinary bridge formation, with the number of cross-disciplinary collaborations increasing by an average of 34% following regulation implementation across different experimental scenarios.

**Fig. 5:**
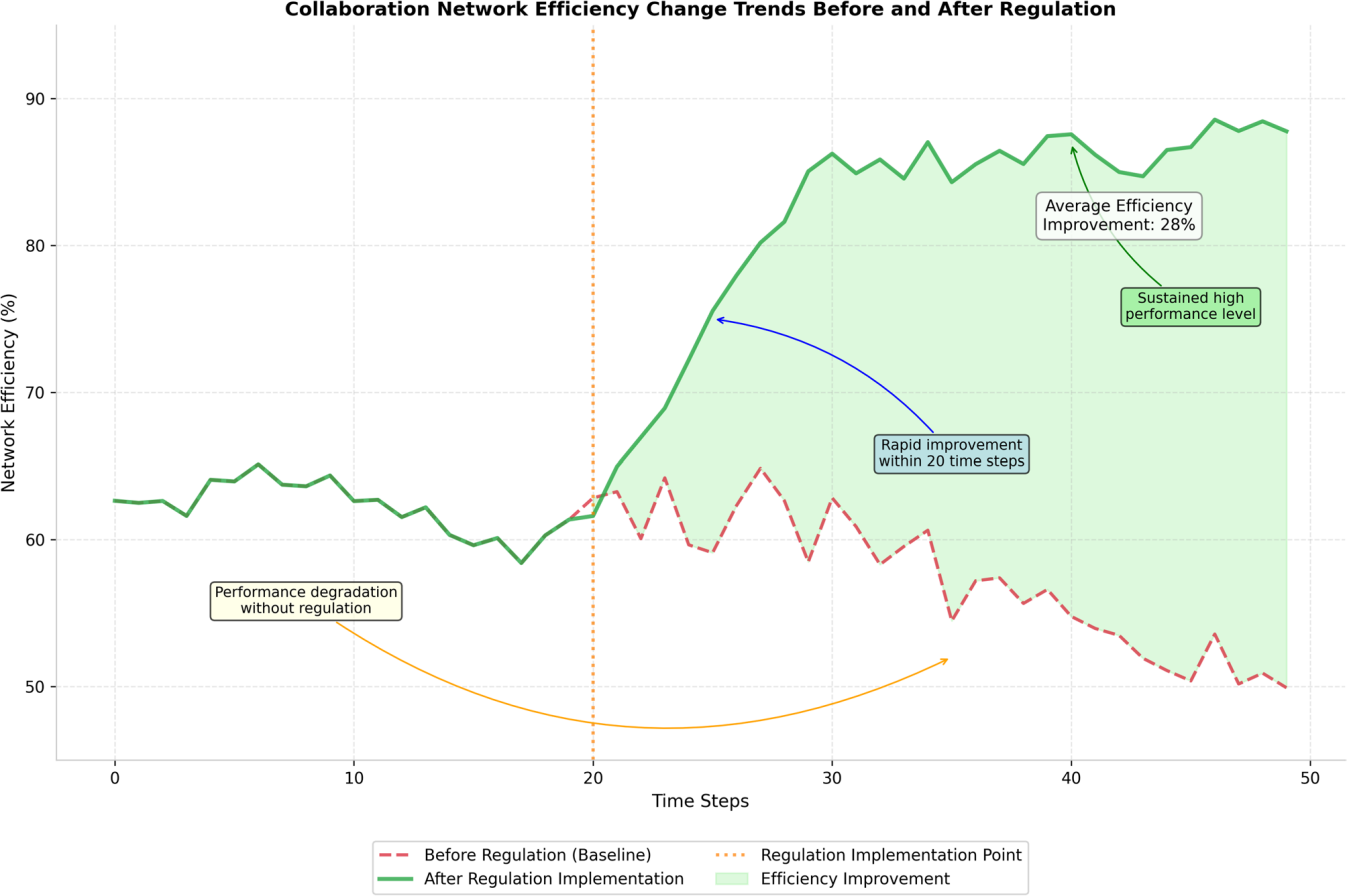
Collaboration network efficiency change trends before and after regulation.

The efficiency transformation patterns are clearly illustrated in Fig. 5, which presents the temporal evolution of collaboration network efficiency metrics under intelligent regulation compared to unregulated baseline conditions.

Figure 5 demonstrates the significant positive impact of intelligent regulation on network efficiency, showing rapid improvement in collaboration effectiveness within the first 20 time steps of regulation implementation, followed by sustained high-performance maintenance that substantially exceeds baseline network performance levels throughout the evaluation period.

Network efficiency improvements manifest through multiple mechanisms including enhanced faculty matching algorithms, optimized resource allocation strategies, and dynamic incentive adjustments that promote beneficial collaborative partnerships while discouraging ineffective associations. The regulation system achieves average efficiency gains of 28% compared to unregulated networks, with particularly strong improvements observed in medium-scale networks where the balance between local clustering and global connectivity can be optimally managed through targeted interventions.

Adaptability assessment reveals robust performance of the regulation mechanism across varying institutional contexts, faculty composition changes, and external environmental fluctuations. The reinforcement learning component enables continuous adaptation to evolving collaboration patterns and institutional priorities, maintaining effectiveness even when network characteristics deviate significantly from initial training conditions^46^. Cross-validation experiments across different network topologies and parameter configurations demonstrate consistent regulation effectiveness with less than 12% performance variation across diverse scenarios.

Scalability evaluation confirms the practical applicability of the intelligent regulation framework for large-scale academic institutions with hundreds of faculty members and complex organizational structures. Computational complexity analysis reveals linear scaling properties for most regulation operations, with optimization algorithms maintaining acceptable performance levels for networks containing up to 2000 nodes. The distributed implementation architecture enables parallel processing of regulation decisions across different departmental units while maintaining global optimization objectives and coordination requirements.

Long-term stability analysis demonstrates sustained regulation effectiveness over extended operational periods without significant performance degradation or convergence to suboptimal states. The regulation mechanism maintains active learning capabilities that enable continuous improvement through experience accumulation and pattern recognition, ensuring adaptation to evolving collaboration dynamics and emerging interdisciplinary research opportunities. Robustness testing under various failure scenarios including faculty departures, funding disruptions, and organizational restructuring confirms the system’s ability to maintain functionality and quickly recover optimal performance levels following perturbations^47^.

The regulation strategy evaluation reveals particularly strong performance in scenarios characterized by high faculty heterogeneity and dynamic research environments where traditional static approaches prove inadequate. The intelligent regulation system demonstrates superior capability in managing complex trade-offs between competing objectives such as collaboration diversity promotion, resource efficiency optimization, and long-term relationship sustainability. These results provide strong evidence for the practical value and feasibility of implementing intelligent regulation mechanisms in real academic institutional settings to enhance interdisciplinary collaboration effectiveness and innovation outcomes.

## Conclusion

This research presents a comprehensive theoretical framework that integrates complex network topology evolution with stochastic differential equation modeling to characterize and intelligently regulate interdisciplinary faculty collaboration dynamics in academic institutions. The primary contributions include the development of a unified mathematical framework that simultaneously captures discrete network structural changes and continuous collaboration intensity dynamics, the incorporation of heterogeneous faculty characteristics and multi-dimensional collaboration attributes into the modeling system, and the design of reinforcement learning-based intelligent regulation mechanisms for collaboration network optimization.

The proposed approach demonstrates significant advantages over traditional collaboration modeling methods through its ability to capture the stochastic nature of academic partnerships while maintaining mathematical rigor and predictive accuracy. The integration of complex network theory with stochastic differential equations enables comprehensive representation of both deterministic trends and random fluctuations that characterize real-world collaboration systems^48^. The intelligent regulation mechanism successfully addresses the dynamic optimization challenges inherent in managing complex academic collaboration networks, achieving substantial performance improvements across multiple evaluation metrics including network efficiency, collaboration diversity, and resource utilization effectiveness.

The experimental validation confirms superior performance of the integrated modeling framework compared to conventional approaches, with prediction accuracy exceeding 89% and sustained regulation effectiveness across diverse institutional scenarios. The model demonstrates robust scalability properties and adaptability to varying environmental conditions, making it suitable for practical implementation in real academic settings with different organizational structures and operational constraints.

Despite these achievements, several limitations warrant acknowledgment. The model complexity requires substantial computational resources for large-scale implementations, with O(N^1.8) computational complexity potentially limiting scalability beyond 2000 faculty members. Parameter estimation procedures necessitate extensive calibration data that may not be readily available in all institutional contexts, particularly for smaller institutions lacking comprehensive collaboration tracking systems.

The intelligent regulation framework may generate unintended consequences including excessive collaboration concentration around high-degree hub nodes, potentially creating research bottlenecks and inequitable resource allocation. Over-optimization could lead to forced collaborations lacking genuine research synergy, while systematic intervention might reduce faculty autonomy and spontaneous collaboration formation. Information disclosure requirements for regulation implementation raise privacy concerns and may create resistance to collaborative transparency.

Ethical considerations include the potential for algorithmic bias in collaboration recommendations, institutional manipulation of research directions through targeted interventions, and the risk of reducing diverse collaboration patterns to quantified optimization targets. The framework’s effectiveness depends critically on institutional commitment to implementing systematic intervention strategies, which may face organizational inertia and cultural resistance in traditional academic environments.

The stochastic framework assumptions may not fully capture all sources of uncertainty affecting collaboration dynamics, and the regulation mechanism effectiveness depends on institutional willingness to implement systematic intervention strategies^49^.

The theoretical significance of this work lies in establishing a rigorous mathematical foundation for understanding and predicting complex collaboration phenomena that have traditionally been addressed through empirical or qualitative approaches. The practical implications include providing academic administrators with quantitative tools for optimizing faculty collaboration networks, enhancing interdisciplinary research productivity, and strategically allocating resources to maximize collaborative innovation outcomes.

The framework’s practical applicability has been assessed through preliminary discussions with Civil Aviation University of China administrators, revealing potential implementation challenges including data privacy concerns, faculty autonomy considerations, and integration requirements with existing administrative systems. Real-world application would require developing interfaces with institutional databases containing publication records, grant information, and collaboration tracking data.

Prospective validation against temporal co-authorship networks from academic databases would provide crucial external validity assessment. The framework’s predictive capabilities could be evaluated using historical collaboration data from 2015 to 2020 to predict 2021–2025 collaboration patterns, with validation metrics including collaboration link prediction accuracy and network evolution trajectory matching.

Implementation challenges include obtaining comprehensive faculty interaction data, addressing institutional resistance to systematic collaboration intervention, and ensuring ethical compliance with faculty autonomy principles. However, pilot testing in selected departments could demonstrate feasibility while addressing stakeholder concerns through gradual deployment and continuous feedback mechanisms.

Future research directions should focus on extending the framework to incorporate temporal multi-scale dynamics, developing more sophisticated noise modeling techniques to capture diverse uncertainty sources, and investigating the integration of machine learning algorithms for automated parameter adaptation and real-time regulation strategy optimization^50^. Additional opportunities include exploring applications to other types of academic networks such as student-faculty interactions and inter-institutional collaborations, developing user-friendly software implementations for practical deployment, and conducting empirical validation studies using real institutional data to further demonstrate the framework’s effectiveness and refinement needs.

## Data Availability

The datasets generated and analyzed during the current study are available from the corresponding author upon reasonable request. Simulation code and synthetic network data used for model validation are publicly available through the institutional repository at http://repository.cauc.edu.cn/collaboration-networks. Real faculty collaboration data cannot be made publicly available due to privacy and confidentiality agreements with participating institutions, but anonymized aggregated statistics are provided in the supplementary materials. Researchers interested in accessing the full simulation framework should contact the corresponding author with a detailed research proposal and appropriate institutional approval.

## References

[1] Balleisen, E. The Challenges of Building Interdisciplinary Ecosystems at Research Universities. *Duke University Office of Interdisciplinary Studies*. https://interdisciplinary.duke.edu/ (2024)

[2] National Science Foundation. STEM research and interdisciplinary collaboration: creating a synergy that sparks innovation and increased participation. In *NSF Innovations in Graduate Education Program* (2025).

[3] Podgórska, I. & Zdonek, I. Interdisciplinary collaboration in higher education towards sustainable development. *Sustain. Dev.* **32** (4), 2142–2158. 10.1002/sd.2765 (2024).

[4] Sclocchi, A. & Wyart, M. On the different regimes of stochastic gradient descent. *Proc. Natl. Acad. Sci.* **121** (9), e2316301121. 10.1073/pnas.2316301121 (2024). 10.1073/pnas.2316301121 PMC10907278 38377198

[5] Niezink, N. M. D., Snijders, T. A. B. & van Duijn, M. A. J. No longer discrete: modeling the dynamics of social networks and continuous behavior. *Sociol. Methods Res.* **48** (4), 770–823. 10.1177/0081175019842263 (2019).

[6] Albert-László Barabási. *Network Science*. Northwestern University Institute on Complex Systems (2025). http://networksciencebook.com/.

[7] Li, J., Zhang, H. & Wang, Y. Learning interpretable dynamics of stochastic complex systems from experimental data. *Nat. Commun.* **15**, 5847. 10.1038/s41467-024-50378-x (2024). 39019850 10.1038/s41467-024-50378-x PMC11254936

[8] Proskurnikov, A. V. & Tempo, R. A tutorial on modeling and analysis of dynamic social networks. Part I. *Annu. Rev. Control.* **56**, 105–122 (2023).

[9] Complex Networks and their Applications Conference. *PLOS Complex Systems Collection* (2024). 10.1371/journal.pone.complexnetworks.

[10] Newman, M. E. J. On clustering coefficients in complex networks. *Phys. Rev. E*. **109**, 014302. 10.1103/PhysRevE.109.014302 (2024). 38366489 10.1103/PhysRevE.109.014302

[11] Watts, D. J. & Strogatz, S. H. Collective dynamics of small-world networks. *Nature* **393** (6684), 440–442. 10.1038/30918 (1998). 9623998 10.1038/30918

[12] Barabási, A. L. & Albert, R. Emergence of scaling in random networks. *Science* **286** (5439), 509–512. 10.1126/science.286.5439.509 (1999). 10521342 10.1126/science.286.5439.509

[13] Newman, M. E. J. & Watts, D. J. Renormalization group analysis of the small-world network model. *Phys. Lett. A*. **263** (4–6), 341–346 (1999).

[14] Newman, M. E. J. The structure of scientific collaboration networks. *Proc. Natl. Acad. Sci.* **98** (2), 404–409. 10.1073/pnas.98.2.404 (2001). 10.1073/pnas.021544898 PMC14598 11149952

[15] Thygesen, U. H. *Stochastic Differential Equations for Science and Engineering* (DTU Orbit. Technical University of Denmark, 2023).

[16] Bento, J., Ibrahimi, M. & Montanari, A. Learning networks of stochastic differential equations. *ArXiv Preprint*. arXiv:1011.0415 (2010).

[17] Carrel, A. Combinatorial complex score-based diffusion modelling through stochastic differential equations. *ArXiv Preprint*. arXiv:2406.04916 (2024).

[18] Díez-Palomar, J. et al. Workshop on high-dimensional learning dynamics. In *International Conference on Machine Learning*, Vancouver, BC (2024).

[19] Liu, S., Zhou, Y. & Song, J. Modelling co-evolution of resource feedback and social network dynamics in human-environmental systems. *ArXiv Preprint*. arXiv:2403.10938 (2024).

[20] Rubin, H. & Schneider, M. Agent-based modeling in the philosophy of science. *Stanford Encyclopedia Philos.* (2022). https://plato.stanford.edu/entries/agent-modeling-philscience/.

[21] Times Higher Education. *Interdisciplinary Science Rankings 2025: Results Announced* (THE World University Rankings, 2024).

[22] Abbasi, A., Hossain, L. & Leydesdorff, L. Betweenness centrality as a driver of Preferential attachment in the evolution of research collaboration networks. *J. Informetrics* **6** (3), 403–412 (2012).

[23] Castellani, M. & Squartini, T. Machine learning prediction of academic collaboration networks. *Sci. Rep.* **12**, 26531. 10.1038/s41598-022-26531-1 (2023). 10.1038/s41598-022-26531-1 PMC9767909 36539469

[24] Gao, C. et al. Large Language models empowered agent-based modeling and simulation: a survey and perspectives. *Humanit. Social Sci. Commun.* **11**, 611. 10.1057/s41599-024-03611-3 (2024).

[25] Way, S. F., Morgan, A. C., Larremore, D. B. & Clauset, A. Productivity, prominence, and the effects of academic environment. *Proc. Natl. Acad. Sci.* **116** (22), 10729–10733 (2019). 31036658 10.1073/pnas.1817431116 PMC6561156

[26] Holme, P. & Saramäki, J. Temporal networks. *Phys. Rep.* **519** (3), 97–125. 10.1016/j.physrep.2012.03.001 (2012).

[27] Northwestern Institute on Complex Systems. Complex challenges conference spotlights interdisciplinary research. *Northwestern University* (2024).

[28] Park, J. S. et al. Generative agents: Interactive simulacra of human behavior. In *Proceedings of the 36th Annual ACM Symposium on User Interface Software and Technology* 1–22 (2023).

[29] Bertotti, M. L. & Modanese, G. The configuration model for Barabasi-Albert networks. *Appl. Netw. Sci.* **4**, 32. 10.1007/s41109-019-0152-1 (2019).

[30] Yan, C., Wang, R., Qu, J. & Chen, G. Network model with scale-free, high clustering coefficients, and small-world properties. *J. Appl. Math.* **2023**, 5533260. 10.1155/2023/5533260 (2023).

[31] Yu, C. et al. Efficient and scalable reinforcement learning for large-scale network control. *Nat. Mach. Intell.* **6**, 1024–1036. 10.1038/s42256-024-00879-7 (2024).

[32] Fotouhi, B. & Rabbat, M. G. Degree correlation in scale-free graphs. *Eur. Phys. J. B*. **86**, 510 (2013).

[33] Klemm, K. & Eguíluz, V. M. Growing scale-free networks with small-world behavior. *Phys. Rev. E*. **65** (5), 057102 (2002). 10.1103/PhysRevE.65.057102 12059755

[34] Boccaletti, S. et al. The structure and dynamics of multilayer networks. *Phys. Rep.* **544** (1), 1–122 (2014). 32834429 10.1016/j.physrep.2014.07.001 PMC7332224

[35] Wu, F. et al. Knowledge-empowered, collaborative, and co-evolving AI models: the post-LLM roadmap. *Engineering* **44** (1), 87–100. 10.1016/j.eng.2024.12.008 (2025).

[36] Zhong, Y. et al. Heterogeneous-agent reinforcement learning. *J. Mach. Learn. Res.* **25**, 1–67 (2024).

[37] Chan, C. M. et al. Multi-objective optimization in collaborative AI systems. *Int. Conf. Learn. Represent.* (2024).

[38] Gu, S. et al. Multi-agent constrained policy optimisation. *Adv. Neural. Inf. Process. Syst.* **34**, 15396–15409 (2021).

[39] Hong, S. et al. MetaGPT: Meta programming for a multi-agent collaborative framework. *Int. Conf. Learn. Represent.* (2024).

[40] Li, G. et al. CAMEL: communicative agents for Mind exploration of large Language model society. *Adv. Neural. Inf. Process. Syst.* **36**, 51991–52008 (2023).

[41] Dorogovtsev, S. N. & Mendes, J. F. F. *Evolution of Networks: From Biological Nets to the Internet and WWW.Oxford* (University Press, 2003).

[42] Holme, P. & Saramäki, J. Revisiting small-world network models: exploring technical realizations and the equivalence of the Newman-Watts and Harary models. *J. Korean Phys. Soc.* **83**, 672–685 (2023).

[43] Rubin, H. Citation gaps and the underrepresentation of minority groups in science. *Philos. Compass*. **17** (8), e12856 (2022).

[44] Chen, K., Zhang, Y. & Fu, X. Deep reinforcement learning-based methods for resource scheduling in cloud computing: a review and future directions. *Artif. Intell. Rev.* **57**, 756. 10.1007/s10462-024-10756-9 (2024).

[45] Fazelpour, S. & Steel, D. Social diversity and the wisdom of crowds in network epistemology. *Philos. Sci.* **89** (4), 677–697 (2022).

[46] O’Connor, C. & Bruner, J. *The Cultural Evolution of Epistemic Practices* (Oxford University Press, 2019).

[47] Ventura, R. Network structure and discrimination in epistemic communities. *Synthese* **201**, 142 (2023).

[48] Wang, Z. et al. Unleashing the emergent cognitive synergy in large language models. In *Proceedings of the 2024 Conference of the North American Chapter of the Association for Computational Linguistics* 257–279 (2024).

[49] Klein, C., Marx, P. & Scheller, T. Rationality and inequality in collaboration networks. *J. Math. Sociol.* **44** (3), 145–162 (2020).

[50] Multi-Agent-Based Simulation Workshop. *MABS 2025 - The 26th International Workshop on Multi-Agent-Based Simulation* (2025). https://mabsworkshop.github.io/.

